# Supplementary material for: Associations of n-3, n-6 Fatty Acids Intakes and n-6:n-3 Ratio with the Risk of Depressive Symptoms: NHANES 2009–2016
Source: Nutrients. 2020 Jan 16;12(1):240. doi: 10.3390/nu12010240 (PMC7019726; doi:10.3390/nu12010240)
Supplement: Supplementary file 1 [file nutrients-12-00240-s001.pdf]

Supplementary Table 1. The classifications of categorical covariates.

Covariates	classifications				
**Age group**	18-44 years,		44-59 years,	≥60years	
**Gender**	Male,		Female		
**Race**	Mexican American,	Other Hispanic,	Non-Hispanic White,	Non-Hispanic Black,	Other race
**Marital status**	Married/Living with partner,		Windowed/divorced/separated/Never married		
**Educational level**	Below high school		High school,	Above high school	
**Household income**	<$20000,	$20000-$44999,	$50000-$74999,	≥75000	
**Body mass index**	< 25 kg/m^2,		25 to <30 kg/m^2,	≥30 kg/m^2	
**Work activity**	Vigorous activity,		Moderate activity,	other	
**Recreation activity**	Vigorous activity,		Moderate activity,	other	
**Smoking at least 100cigarettes in life**	Yes,		No		
**Have at least 12 alcohol drinks/years**	Yes,		No		
**Hypertension**	Yes,		No		
**Diabetes**	Yes,		No		
**Coronary heart disease**	Yes,		No		
